# Sensor Data Fusion for a Mobile Robot Using Neural Networks

**DOI:** 10.3390/s22010305

**Published:** 2021-12-31

**Authors:** Andres J. Barreto-Cubero, Alfonso Gómez-Espinosa, Jesús Arturo Escobedo Cabello, Enrique Cuan-Urquizo, Sergio R. Cruz-Ramírez

**Affiliations:** 1 Tecnologico de Monterrey, Escuela de Ingenieria y Ciencias, Av. Epigmenio González 500, Fracc. San Pablo, Querétaro 76130, Mexico; A01208643@itesm.mx (A.J.B.-C.); arturo.escobedo@tec.mx (J.A.E.C.); ecuanurqui@tec.mx (E.C.-U.); 2 Tecnologico de Monterrey, Escuela de Ingenieria y Ciencias, Av. Eugenio Garza 10 Sada 300, Lomas del Tecnológico, San Luis Potosí 78211, Mexico; rolando.cruz@tec.mx

**Keywords:** sensor data fusion, mobile robot, artificial neural network, improved LiDAR, occupancy grid map

## Abstract

Mobile robots must be capable to obtain an accurate map of their surroundings to move within it. To detect different materials that might be undetectable to one sensor but not others it is necessary to construct at least a two-sensor fusion scheme. With this, it is possible to generate a 2D occupancy map in which glass obstacles are identified. An artificial neural network is used to fuse data from a tri-sensor (RealSense Stereo camera, 2D $360{}^{{}^{\circ}}$ LiDAR, and Ultrasonic Sensors) setup capable of detecting glass and other materials typically found in indoor environments that may or may not be visible to traditional 2D LiDAR sensors, hence the expression improved LiDAR. A preprocessing scheme is implemented to filter all the outliers, project a 3D pointcloud to a 2D plane and adjust distance data. With a Neural Network as a data fusion algorithm, we integrate all the information into a single, more accurate distance-to-obstacle reading to finally generate a 2D Occupancy Grid Map (OGM) that considers all sensors information. The Robotis Turtlebot3 Waffle Pi robot is used as the experimental platform to conduct experiments given the different fusion strategies. Test results show that with such a fusion algorithm, it is possible to detect glass and other obstacles with an estimated root-mean-square error (RMSE) of 3 cm with multiple fusion strategies.

## 1. Introduction

Sensor data fusion is a crucial task to process information from a multiple sensor set up in a mobile robot [1]. Multiple distance measurement devices with different sensing technologies (2D Laser Imaging Detection and Ranging (LiDAR), Stereo Camera, and Ultrasonic Sensors) in the same robotic platform allow the detection of a wider range of obstacle types. However, this approach requires a computationally expensive (for a mobile robot central processing unit (CPU)) data fusion algorithm to merge the distance readings from all sensors into a single reliable and accurate distance to obstacle measurement.

Sensor data fusion for mobile applications is varied, but in most cases, the approach used will be the determining factor regarding the outcome quality of the system. It is also the most differentiating factor between fusion strategies [2]. Outcome quality can be determined by comparing real-time capability, RMSE of the data output, physical implementation, and reproducibility of the experimental platform. Fusion strategies can be based on: probabilistic approach such as Factor graphs [3], extended Kalman Filters [4], Bayesian methods [5], and Particle Filters [6] or it can be based on artificial intelligence such as Neural Networks (NN) [7] or Fuzzy Logic [8,9]. Some of the most common approaches are Kalman Filters and NN with a wide range of implementation configurations [10,11,12]. Extended Kalman Filters [13] were used by Dobrev, Gulden, and Vossiek to improve an indoor positioning system using multi-modal sensor fusion for service robots’ applications. This implementation requires a fixed in placed sensor infrastructure and laser scanner measurement errors were up to 30 cm in the presence of glass doors. Jing et al. [14] implemented a multi-sensor data fusion method for fault diagnosis of a planetary gearbox. They fused vibration, acoustic, electric current, instantaneous angular speed signals and were able to learn the features at different fusion levels using Deep Convolutional Neural Networks (DCNN), Back Propagation Neural Networks (BPNN), and a support vector machine decision-making algorithm. While this comprehensive report shows better results for DCNN, BPNN is not far behind in the data fusion level. A data fusion contest [15] for processing High-Resolution LiDAR and RGB data of outdoor aerial photography involving water, trees, cars, and boats shows that the complement of RGB and LiDAR with Deep Neural Networks (DNN) was the best approach. Hua et al. [16] used a Laser Range Finder (LRF) and a digital camera as data generators for an Artificial Neural Network (ANN) with a standard backpropagation algorithm that outputs the speed and steers values for the navigation of an intelligent wheelchair. Their results are consistent with the previous state of the art related work. The main cause for measurement error in mobile navigation and localization is the inability to detect certain obstacles due to the physical nature of the obstacle. Mancini et al. [17] implemented an embedded multisensory system for a safe point-to-point navigation system where a LiDAR and dual sonars were tested at detecting obstacles. Their experiments show that by using an estimation of threat algorithm, they can detect more obstacles with the fusion strategy than the individual sensor’s output. Buonocore et al. [6] used three sensing technologies: a vision sensor, an infrared sensor, and a sonar sensor, which were fused using an extended Kalman Filter on the occupancy grid maps generated. Li et al. [18] used a fiducial feature extraction to fuse a 3D occupancy map out of a 2D LiDAR and a camera data streams. The authors of [19] propose an approach for autonomous navigation of mobile robots in faulty situations where the main objective is to extend the fault tolerance strategy to simultaneous localization and mapping in presence of sensor or software faults in the data fusion process. The proposed approach is based on the extended Kalman filter for simultaneous localization and mapping when an absolute localization sensor is available, and the experiments are performed employing an omni-drive mobile robot, equipped with embedded sensors, namely: wheel encoders, gyroscope, two laser rangefinders, and an external sensor for the absolute position (indoor global positioning system). The obtained results demonstrate the effectiveness of the proposed approach where it was found that its fault tolerance performance is based essentially on the selected residuals and the values of the fault detection thresholds to be used for fault detection and isolation. Sasiadek and Hartana [20] present the data fusion system for mobile robot navigation, where the odometry and sonar signals are fused using Extended Kalman Filter (EKF) and Adaptive Fuzzy Logic System (AFLS). The AFLS was used to adapt the gain and therefore prevent the Kalman filter divergence. The fused signal is more accurate than any of the original signals considered separately. The enhanced, more accurate signal is used to guide and navigate the robot. The authors of [21] propose a holonomic mobile platform to detect and follow targets, moving on the ground, in an indoor environment. Using multi-sensor data flow, the control algorithm can localize the target object by its shape and follow it. The robot will maintain the desired orientation and distance while following the target. Additionally, the mobile platform has also a fully manual control mode option and is able to recognize human gesture commands. Finally, a novel approach presented by Tibebu et al. [22] uses the variation of range measurements between neighboring pointclouds using a two-step filter.

In this article, a 2D LiDAR system was developed with the improved capability to detect glass and other obstacles in a 3D environment by fusing data between three main sensing technologies in a mobile robot platform. We seek to increase the number of obstacle materials that the mobile robot can detect with the same mapping run without adding infrastructure to the working environment. Thus, decreasing the error in navigation and localization of the robot. Our sensor setup can detect a wide range of surfaces at different heights such as glass, concrete, wood, aluminum among others regardless of the interior lighting condition. The first step in the data fusion consists of preprocessing the raw sensor signals to avoid extreme outliers in the dataset. This issue is particularly common with the ultrasonic sensors, for which a Kalman Filter was implemented [4,23]. The data generated from the LiDAR and the stereo camera are preprocessed to discard the distance measurements outside of the FoV (Field of View) of the stereo camera. Secondly, a homogeneous transformation matrix is computed using the ROS infrastructure to make sure the obstacles appear in the same direction to all sensors. The third step consists of generating the data to train the neural network. Fourth, the neural network with an ADAM Optimizer as training algorithm [24], and a Rectified Linear Unit (ReLu) activation function which is reported in [25,26] to have good behavior on non-linear data. The weights of the trained NN are saved as a *.h5* file with the main structure of the network so that it can be imported and used in real time processing. Three field experiments were conducted to verify the RMSE of the distance measurements with the presence of glass, and other obstacles and compare a LiDAR-Camera-Sonar (LCS) fusion strategy with a LiDAR-Sonar (LS) fusion strategy and a sole LiDAR strategy. Results show that for both fusion strategies the RMSE is between two and three cm while the individual LiDAR readily available and most common approach for SLAM presents an RMSE of over two meters.

The rest of the paper is composed of the materials and methods with the main concepts in Section 2 and the experimental setup in Section 3. The results are in Section 4 and conclusions in Section 5.

## 2. Materials and Methods

Data fusion strategies can be classified according to different criteria such as architecture, input-output structure, type of data to fuse, among others. In this case, we have a centralized architecture with a redundant approach and distance data as input and output. Given the varied nature of the working principles for the sensors that function as the input data source, different processing methods were required to start the data fusion process.

### 2.1. Ultrasonic Sensor

**Figure 1:**
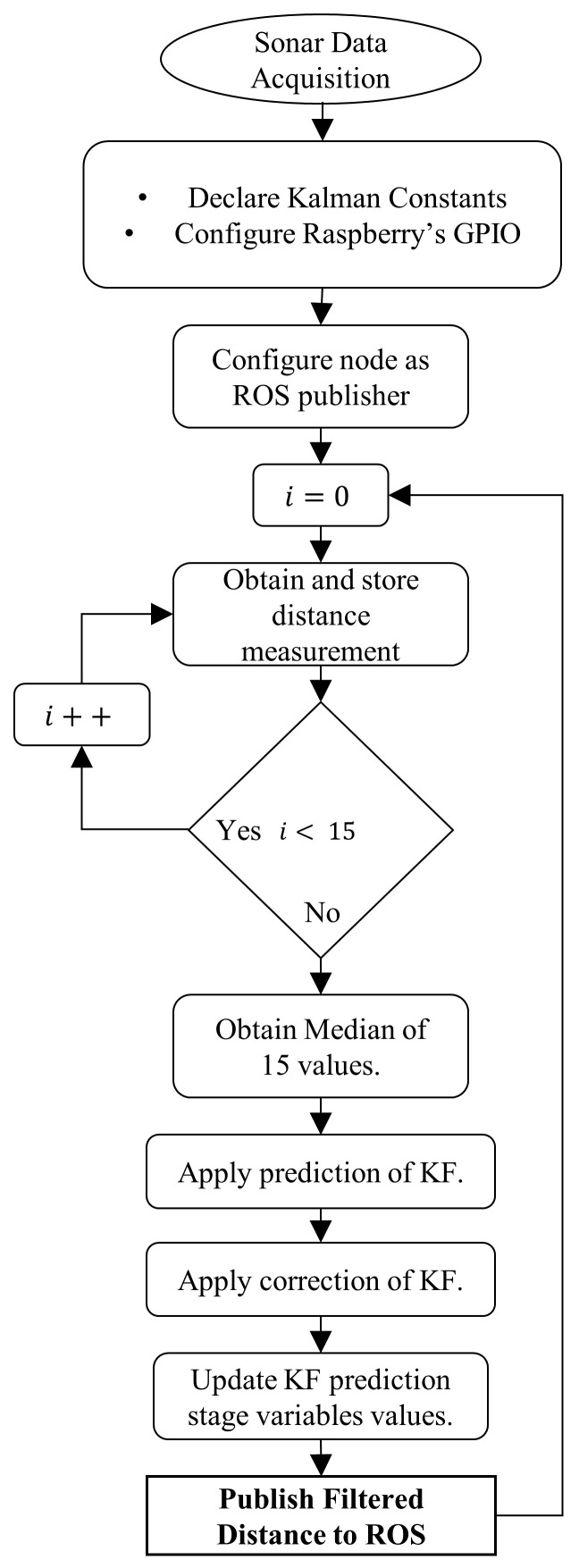
Ultrasonic sensor data acquisition flowchart and filtering strategy.

The ultrasonic sensor or sonar is one of the most common inexpensive types of sensors for mobile robots. Its principle of operation relies on the speed of the sound wave. This type of sensor suffers from high dispersion values between measurements. A preprocessing stage with both a median and a Kalman filter are applied to the ultrasonic sensor measurements. The median filter removes sudden spikes in measurement values without upsetting the Kalman Filter stabilization stage [27]. Abundant Kalman Filter implementations for sensing devices are presented by [4,27,28] which present a reference guide as to the numeric constant variables required to initialize the filter. However, these values need to be manually adjusted to achieve the desired behavior of the filter. The data acquisition strategy and workflow are presented in Figure 1. From Figure 1 we can conclude that a total of 15 measurements are taken before applying the median filter or the Kalman Filter, thus improving measurement accuracy.

Since the ultrasonic sensor does not have angular resolution, that is, only one measurement regardless of the position of the obstacle within the range cone. The same distance measurement is applied to all the angular positions of the range cone for that specific sensor.

#### Kalman Filter

The discrete Kalman Filter algorithm used can perform the recursive estimation of the state of the dynamic behavior of a system even when the model of the dynamic system is not well known [27]. So, the algorithm formulation is designed to work in time intervals. The algorithm itself has two stages: prediction (Equations (1) and (2)) and correction (Equations (3)–(5)). In the prediction stage ${\hat{x}}_{k}{}^{\left(-\right)}$ is a priori state estimated for the posteriori state ${\hat{x}}_{k}$ and $P_{k}{}^{\left(-\right)}$ is the covariance matrix a priori of the error estimation. Q is the process covariance matrix. In all the Kalman Filter equations, the symbol (−) refers to “a priori” state.

(1) $${\hat{x}}_{k}{}^{\left(-\right)}=F{\hat{x}}_{k-1}+Bu_{k-1}$$

(2) $$P_{k}{}^{\left(-\right)}=FP_{k-1}F^{T}+Q$$

The correction phase of the filter aims to minimize the covariance matrix error $P_{k}$ by computing the Kalman gain $K_{k}$, updating the state estimation value with the measurement vector $z_{k}$. R is the covariance value of the measurement noise.

(3) $$K_{k}=P_{k}{}^{\left(-\right)}H^{T}{\left(HP_{k}{}^{\left(-\right)}H^{T}+R\right)}^{-1}$$

(4) $${\hat{x}}_{k}={\hat{x}}_{k}{}^{\left(-\right)}+K_{k}\left(z_{k}-H{\hat{x}}_{k}{}^{\left(-\right)}\right)$$

(5) $$P_{k}=P_{k}{}^{\left(-\right)}(I-K_{k}H)$$

In Table 1 the numerical constants in the Kalman filter algorithm are employed along with the fine-tuned Q and R constants. Numerical values from Table 1 are substituted in Equations (1)–(5) and both stages are executed continuously to generate the estimated value of the measurement. Variable ${\hat{x}}_{k}{}^{\left(-\right)}$ and $P_{k}{}^{\left(-\right)}$ are declared initially with an arbitrary value to function as the initial value of the state estimation. With each update run, these variables acquire a more accurate state estimation.

### 2.2. Stereo Camera

Stereo or 3D Cameras operate using the same principle as the human eye. In which, there are two individual lenses separated by a known distance. Depth information is found by calculating disparities between the images from the two visual points.

The stereo camera provides its data in pointcloud or sparse data format. To correctly interpret the relevant information of the image, a conversion between sparse to dense data format is in place. So, the XYZ coordinates need to be extracted using ROS numpy infrastructure. In this way, each detected pixel in the image has a numerical distance coordinate assigned to it. From the XYZ points, we perform a 3D to 2D simple orthographic parallel projection, where we only use the coordinates that represent an obstacle to the robot (From the ground to 1 cm above the LiDAR. By doing this, we discard not useful information and reduce the number of points to transform to a fraction of the original value.

A simple parallel orthographic projection onto the plane *z* can be defined as:

(6) $$P=\left[\begin{array}{ccc} 1 & 0 & 0\\ 0 & 1 & 0\\ 0 & 0 & 0 \end{array}\right]\;$$

For each point $v=\left(V_{x},\;V_{y},\;V_{z}\right)$ we project from the 3D space, the transformed point *Pv* in a 2D space is:

(7) $$Pv=\left[\begin{array}{ccc} 1 & 0 & 0\\ 0 & 1 & 0\\ 0 & 0 & 0 \end{array}\right]\left[\begin{array}{c} v_{x}\\ v_{y}\\ v_{z} \end{array}\right]=\left[\begin{array}{c} v_{x}\\ v_{y}\\ 0 \end{array}\right]\exists \;3\;\mathrm{cm}\geq v_{z}\geq -8\;\mathrm{cm}\;$$

**Figure 2:**
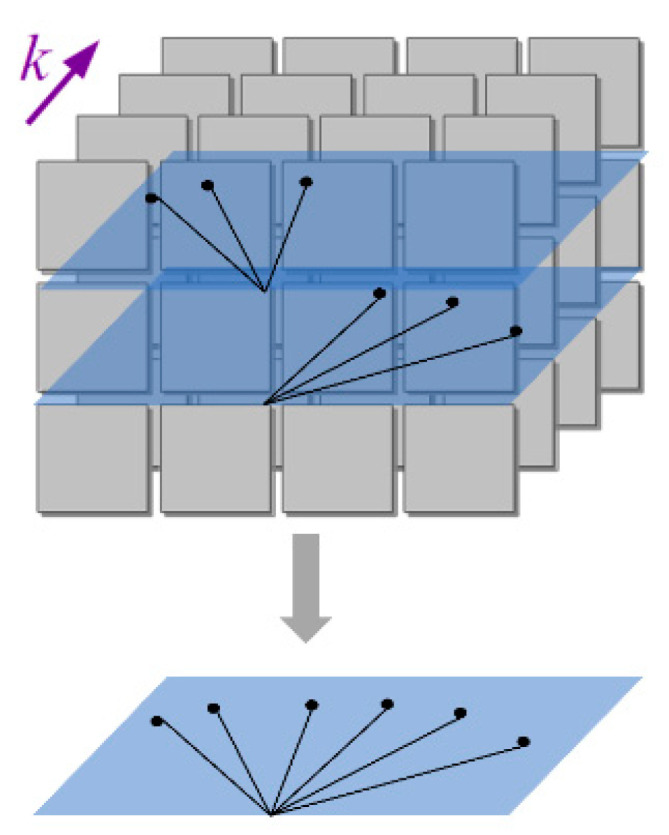
3D to 2D parallel orthographic projection.

A visual representation of point projections in different planes can be analyzed in Figure 2.

### 2.3. LiDAR

The LiDAR technology also relies on the time of travel approach to detect the distance to the nearest obstacle. Moreover, since the emitting source is a beam of light, the sensor is very accurate at detecting the direction of the obstacle if the material of the obstacle does not refract the light beam. Additional adjustments are in place to the LiDAR data stream. Instead of measuring all $360{}^{{}^{\circ}}$ in a 2D configuration, only points facing in the same direction as the camera and sonar will be considered for the fusion strategy. The remainder of the points are directly passed on to complete the construction of the OGM without being fused.

### 2.4. Homogeneous Transformation Matrices

Since all the sensors from Section 2.1, Section 2.2 and Section 2.3 transmit the data w.r.t their respective coordinate frames, a network of homogeneous transformation matrices needs to be computed to the “*base_link*” (Principal) frame of the Turtlebot3. The rotation and translation transformations numerical values were obtained with manual measurements of the sensor placement in the robot. These are accessed in the ROS ecosystem to ensure all sensors are measuring w.r.t the LiDAR reference frame.

As with almost any robot with more than 1 degree of freedom, homogeneous transformation matrices can be used to represent a point w.r.t another reference frame. In our case, allowing the data from all the different sensors to be numerically represented w.r.t the LiDAR reference frame, even though they are all physically placed in different positions. These calculations are possible by following the Special Euclidean Group SE in Equation (8), states:

(8) $$T=\left[\begin{array}{cc} R & p\\ 0 & 1 \end{array}\right]=[\begin{array}{cccc} r_{11} & r_{12} & r_{13} & p_{1}\\ r_{21} & r_{22} & r_{23} & p_{2}\\ r_{31} & r_{32} & r_{33} & p_{3}\\ 0 & 0 & 0 & 1 \end{array}]\exists \;R\in SO\left(3\right),\;P\epsilon R^{3}\;$$

where the *R* matrix represents the rotation between two different frames and the *p* vector represents the direction of the translation between the two different frames. Together they form the homogeneous transformation matrix. The rotation matrix changes depending on the axis around which the rotation takes place.

(9) $$R_{x}\left(\alpha \right)=\left[\begin{array}{ccc} 1 & 0 & 0\\ 0 & \cos\;\alpha & -\sin\;\alpha \\ 0 & \sin\;\alpha & \cos\;\alpha \end{array}\right]\;$$

(10) $$R_{y}\left(\beta \right)=\left[\begin{array}{ccc} \cos\;\beta & 0 & \sin\;\beta \\ 0 & 1 & 0\\ -\sin\;\beta & 0 & \cos\;\beta \end{array}\right]$$

(11) $$R_{z}\left(\gamma \right)=\left[\begin{array}{ccc} \cos\;\gamma & -\sin\;\gamma & 0\\ \sin\;\gamma & \cos\;\gamma & 0\\ 0 & 0 & 1 \end{array}\right]$$

Note, how the Euler angles change depending on the axis around which the rotation takes place in Equations (9)–(11).

The Turtlebot3 robot has a *base_link* reference frame which serves as a reference frame for all the sensors attached to the robot. So, later within the ROS infrastructure, a homogeneous transformation matrix between sensors can be automatically calculated by the *tf2_ros* node of type *static_transform_publisher*. Find the homogeneous transformation matrices with respect to the *base_link* in Equations (9)–(11) where all displacements are in meters and all rotations are in radians.

(12) $$T_{baselink\_rightSonar}=\left[\begin{array}{cccc} 0.9855847 & 0.1691824 & 0 & 0.085\\ -0.1691824 & 0.9855847 & 0 & -0.08\\ 0 & 0 & 1 & 0.102\\ 0 & 0 & 0 & 1 \end{array}\right]$$

(13) $$T_{baselink\_leftSonar}=\left[\begin{array}{cccc} 0.9855847 & -0.1691824 & 0 & 0.085\\ 0.1691824 & 0.9855847 & 0 & 0.08\\ 0 & 0 & 1 & 0.102\\ 0 & 0 & 0 & 1 \end{array}\right]$$

(14) $$T_{baselink\_D435RealSense}=\left[\begin{array}{cccc} 0 & -1 & 0 & 0.06\\ 0 & 0 & -1 & 0.015\\ 1 & 0 & 0 & 0.12\\ 0 & 0 & 0 & 1 \end{array}\right]$$

Since the LiDAR sensor is already attached to the robot in the factory specified position, and the transformation information is embedded in the turtlebot3 libraries, its transformation matrix will be omitted.

### 2.5. Data Fusion

The data fusion most accepted definition is the Joint Directors Laboratory, which states that data fusion is a “multi-level process dealing with the association, correlation, combination of data and information from single and multiple sources to achieve refined position, identify estimates and complete and timely assessments of situations, threats and their significance” [1]. From this general definition, we can conclude that data fusion is a combination of data from single or multiple sources to obtain financially less expensive, higher quality, and more relevant information [2]. Even though a multiple sensors setup may indicate a higher cost compared to a single sensor setup, a 3D LIDAR can cost more than two times the combined cost of a 2D LIDAR, a digital camera, and an ultrasonic sensor altogether. Also, each sensor works using a specific working principle to detect certain materials in each environment. LiDARs are known to have difficulties locating glass and other surfaces in general where the light beam of the LiDAR refracts and does not return to the light emitter. This issue is corrected by adding ultrasonic sensors which can detect glass and refractive surfaces. However, the sonar does not have the angular resolution nor a good enough FoV to detect other obstacles that may be closer to the robot. So, we intend to exploit the best features of a specific sensor and use them to compensate for the drawbacks of the other sensor.

The methods and techniques employed to fuse data are diverse, so to organize them, it is possible to review them using the criteria shown in Table 2. The method used depends on the type of data to be fused and the computational capacity of the hardware where the fusion algorithms run, so it depends on the application.

Given the relationship between the input data sources, the type of fusion that best applies is redundant, since we want to measure the same variable (distance) using multiple sensors which will generate multiple datasets. We use raw data as input in our fusion approach since the first type of data to enter our model will not have any pre-processing done to it, other than the sensor drivers and plugins that convert the physical variable (Light or Sound) onto an electrical pulse. The output “fused” data can be considered a signal, although they are of the same units and data type as the input.

With a centralized data fusion architecture, the data for each sensor are preprocessed. Later, we align the information (Perform the homogeneous transformations) of the pose of each sensor with respect to the LiDAR. In the association section, we arrange the data and make it compatible with the neural network algorithm. Finally, in the estimation section, the NN estimates the real distance to the obstacle given the distance data for each sensor in the fusion working area.

### 2.6. Deep Feed forward Neural Networks

A DFFNN is a type of ANN that provides a powerful solution when trying to create an approximate model of a dataset capable of predicting an output given several inputs. Although a similar approximation can be made with a linear regression system, there are several constraints that the data needs to comply with to have a small RMSE [29]. The ANN can solve complex problems in various fields such as solving function approximations and generation of meaningful patterns [30].

**Figure 3:**
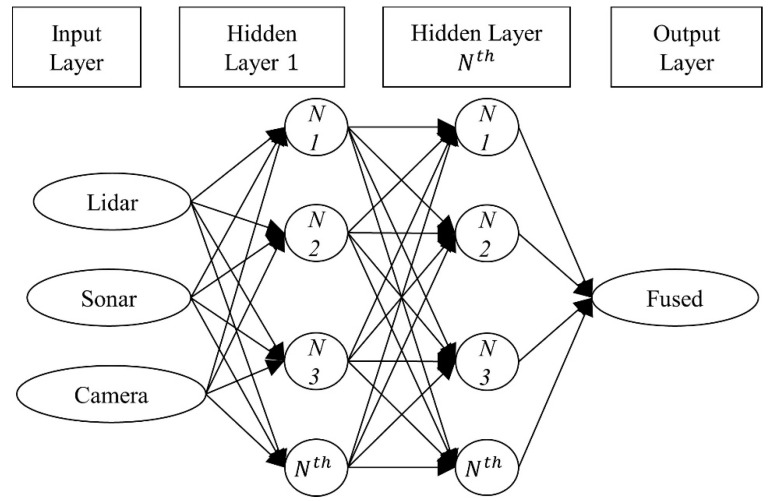
Basic structure of the DFFNN used where each input corresponds to the distance data of each sensor per degree.

In Figure 3 we have the basic structure of a Deep Feed Forward neural network with three input neurons, multiple hidden layers with multiple neurons, and a single output layer. The number of internal neurons on the hidden layer does not have to be of the same dimension as the input layer. The more complicated to analyze or diverse the dataset is, the more neurons are required in the hidden layers. Sometimes, multiple hidden layers with several dozen neurons are necessary to accurately represent a dataset [31].

A Deep Feed Forward neural network is used since the complexity of the problem requires multiple inputs neurons and multiple hidden layers with many neurons per layer. This type of network was revitalized and used extensively from the 90′s onward, due to multiple breakthroughs in the training procedures and algorithms used to update the internal weights of each neuron.

Since ANNs are based on the biological structure of neurons in the brain, computations can be parallelized by adding hidden layers and being able to comprehend multiple complex structures. Each neuron represents a computational node, which activates when the input is strong enough and crosses a defined threshold [31]. The mathematical expression that handles the activation of the neuron is called the Activation Function. There are multiple activation functions widely used for linear and non-linear datasets. The type of function to use is highly dependent on the data structure of the specific application since the mathematical behavior of the function is different in each case scenario. *ReLu* activation function in Equation (15). If the input is positive, the output will be the input. If the input is negative, the output will be zero.

(15) $$g\left(x\right)=\{\begin{array}{c} 0,\;\;x<0\\ x,\;\;x\geq 0 \end{array}$$

The output generated by each neuron is calculated as the weighted sum of the weights of each input link. This value is calculated by:

(16) $$f\left(x\right)=w_{0}\sum_{i=1}^{n}w_{i}x_{i}\;$$

Then, each node will apply a ReLu activation function to the input f(x).

**Figure 4:**
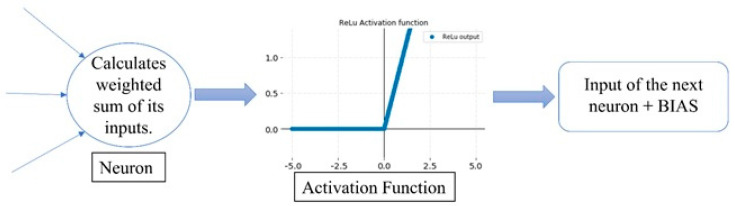
Working principle of the DFFNN at a neuron level.

The estimation section of the data fusion strategy is conducted using an ANN algorithm with one input per sensor, multiple hidden layers, and an output neuron with the “determined distance” to each direction as shown in Figure 4. The NN was trained using an ADAM optimization algorithm and the activation function of choice for each layer is ReLu. The performance of the NN is measured using the RMSE value.

#### 2.6.1. Gradient Stochastic Descent Optimizer

The weight value of each link in the neural network dictates how well trained the network really is. In fact, by training the network, we adjust the weights so that they reflect the expected output more accurately by giving a lower score to the less significant values and a higher score to the more relevant ones.

**Figure 5:**
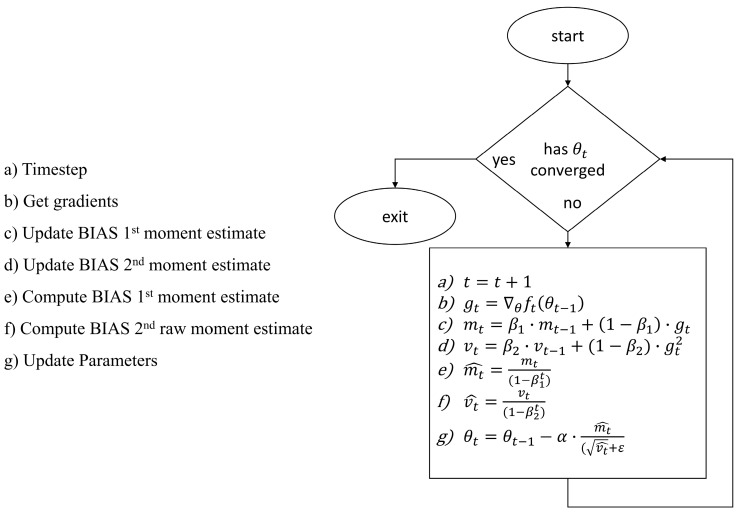
ADAM optimization algorithm workflow.

The Adam optimizer is a newly developed algorithm for first-order gradient-based optimization of stochastic objective functions. It is based on adaptive estimates of lower order moments [32]. The goal of Adam is to find a set of parameters that minimize mean squared error, so it works with sparse gradients and naturally performs a form of step size annealing, see Figure 5. Chang [31] experiments with the effects of using different optimizers on a neural network and concludes that the Adam optimizer is simple to implement, is computationally efficient, and has small memory requirements so it works well in practice and has advantages over other stochastic optimization methods [24].

#### 2.6.2. Neural Network Configuration

Since ANNs are based on the biological structure of neurons in the brain, computations can be paralleled by adding hidden layers and being able to comprehend multiple complex structures. Each neuron represents a computational node, which activates when the input is strong enough and crosses a defined threshold. There are multiple activation functions widely used for linear and non-linear datasets. The type of function to use is highly dependent on the data structure of the specific application since the mathematical behavior of the function is different in each case scenario.

Given the sparse nature of the data, a decay step was implemented to adjust the learning rate as the training through each epoch progressed. The input layer and all the hidden ones of the network are implemented using the *kernel_initializer* “*he_uniform*” as it presents solid results at preventing layer activation outputs from exploding or vanishing during a forward pass through the network. Also, a matrix of BIAS filled with zeros was implemented at each layer. The shuffle parameter was turned on to stop the network from memorizing and encourage it to learn. The batch size was set to 32 initially but had to be dropped as part of the tuning step.

A small validation step was implemented when generating the NN model. This allows testing for different tuning parameters without having to run time-sensitive data through the network.

### 2.7. Occupancy Grid Map

An OGM as its name implies is a map that shows the obstacles and the empty space of a given environment. It is particularly useful in robotics, where it serves as a guideline for robots to perform navigation tasks. Usually, the white area around the robot represents empty space. Black and green dots represent an occupied grid (obstacle), and light gray represents unknown space.

Typically, OGM is constructed using Simultaneous Localization and Mapping in conjunction with one of several grid mapping techniques. Some of these techniques have been implemented in ROS as standalone packages such as Hector SLAM and Gmapping. Given the good results reported by Grisetti, Stachniss, and Burgard [33] and how well-known this method is among the community, we use *Gmapping* to generate our OGM.

## 3. Experimental Setup

### 3.1. Hardware

**Figure 6:**
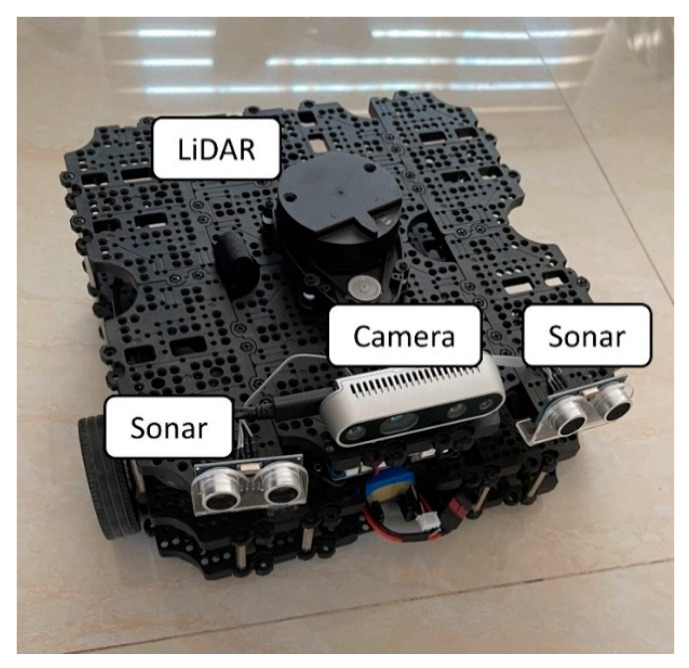
Sensor’s physical placement on Turtlebot 3 Waffle Pi.

The main experimental setup consists of the Turtlebot3 Waffle Pi by Robotis^®^ running Ubuntu Server 20.04.1 on a Raspberry Pi 4 8 Gb and a Toshiba Satellite L845 Laptop running Ubuntu 20.04 Desktop. The LiDAR used is a Robotis LDS-01 2D $360{}^{{}^{\circ}}$ which uses an LDS2USB module as a data adapter to easily connect the LiDAR to the raspberry. The ultrasonic sensors are HC-SR04, connected to a voltage divider so that the 5V at which the sensor operates does not damage the 3.3 V tolerant pins on the raspberry. The stereo camera is connected to the raspberry via a USB-C to USB 3.0 interface. Given the power consumption nature of the devices involved, a fully charged 1800 mAh LiPo battery is enough for ~20 min of usage. The stereo camera and ultrasonic sensors were added to the Turtlebot3 in strategic positions so that each sensor has an unobstructed FoV as shown in Figure 6. Occupying the central position on top of the robot is the LiDAR. The ultrasonic sensors are in the front right and left corners of the robot. Lastly, the RealSense camera with the aluminum enclosure is placed in the middle front section of the robot.

**Figure 7:**
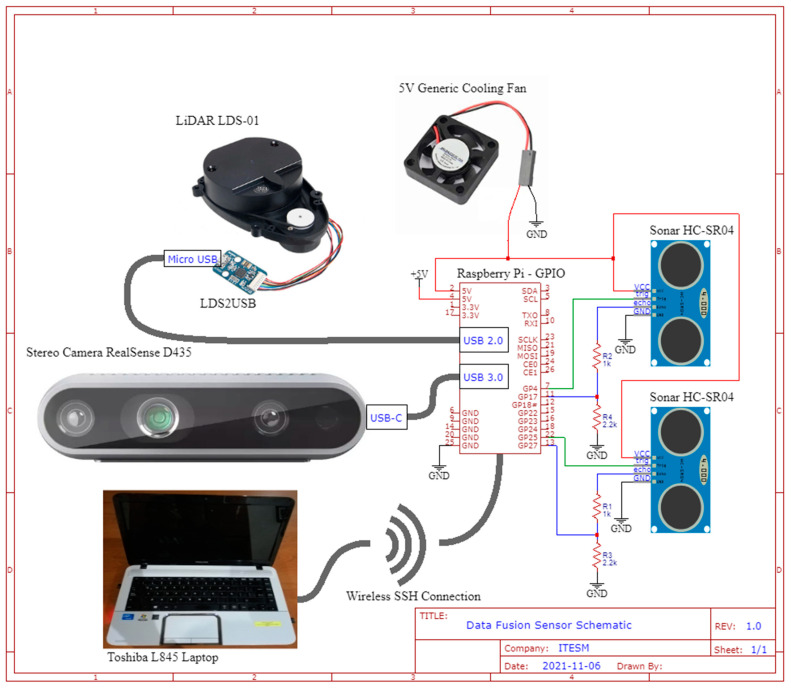
Sensor’s electrical connections schematic to the Raspberry Pi.

The connections between the LiDAR and the Raspberry are via a USB2LDS adaptor. The camera is connected directly to one of the USB 3.0 connectors in the raspberry. The ultrasonic sensors are connected to the raspberry via the GPIO pins, via a voltage divider since the raspberry is not 5 V tolerant on the GPIO pins and the ultrasonic sensor is. The basic circuitry can be easily analyzed from Figure 7. As for the rest of the electrical setup of the robot, it can be found on the official Turtlebot3 by Robotis^®^ website.

The stereo camera is mechanically attached to the robot via a 0.25 in by 0.75  in bolt which screws to the bottom of the robot frame. The ultrasonic sensors are placed in acrylic mountings designed specifically for this sensor. The sensor mount is screwed to the chassis of the robot.

### 3.2. Proving Ground

**Figure 8:**
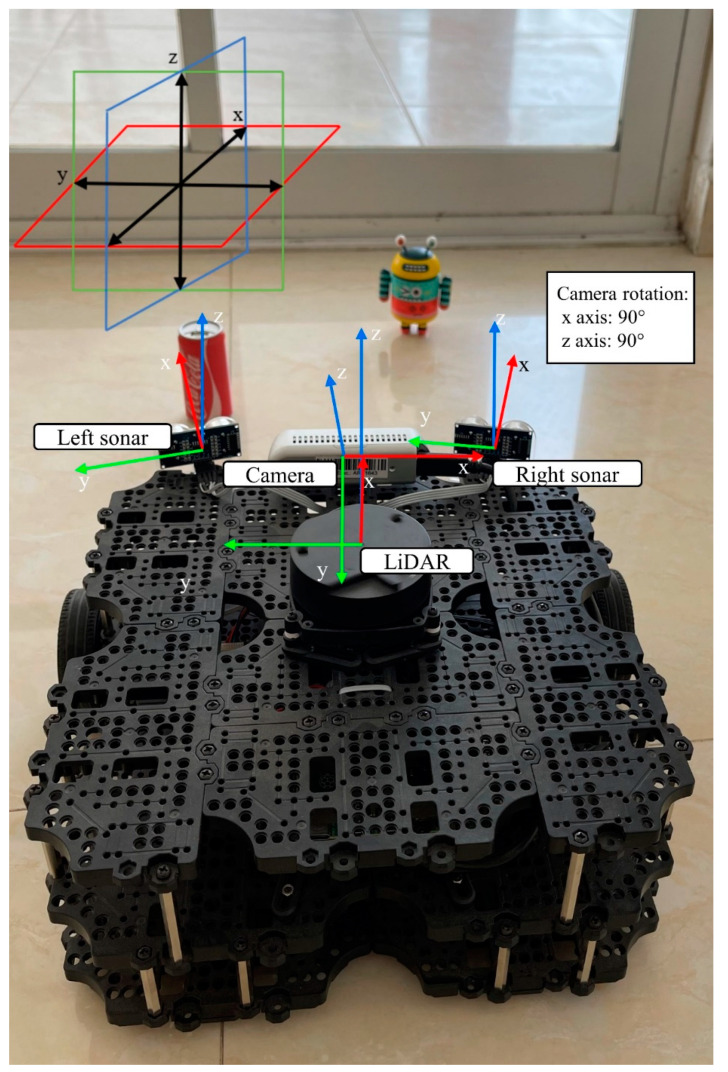
Sensors’ reference frames with obstacle in proving ground.

**Figure 9:**
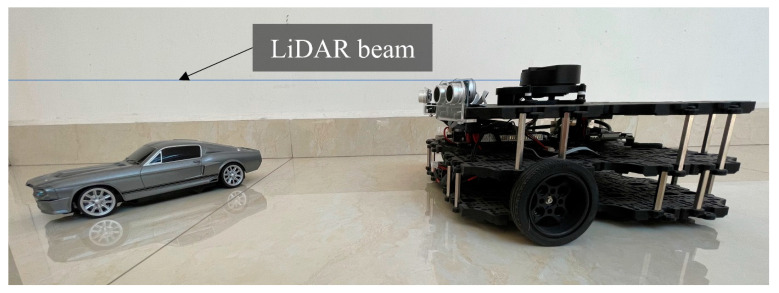
Sole 2D LiDAR beam does not detect shorter obstacles underneath it.

The experiments take place in an indoor environment with significant amounts of glass panels and objects present underneath the FoV of the LiDAR, also shown in Figure 8 and Figure 9.

Since the speed of sound changes with temperature, the room temperature is measured and taken into consideration when calculating the distance from the Time of Travel of the sound wave. The ultrasonic sensor used has an FoV of $30{}^{{}^{\circ}}\pm 6{}^{{}^{\circ}}$, so a second sensor will be installed to cover the second half of the surface area the first ultrasonic sensor does not cover. The Stereo Depth Camera has a theoretical limited horizontal FoV of $87{}^{{}^{\circ}}\pm 3{}^{{}^{\circ}}$, so all sensors will be limited to measure the same surface area as the camera.

The camera resolution was changed from 1920×1080 to 680×460 to decrease the size of the resulting image and improve the processing time.

### 3.3. Software

**Figure 10:**
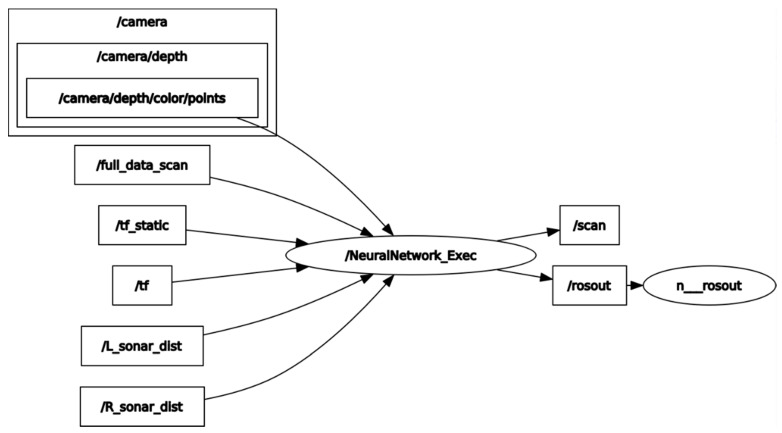
ROS node topology contribution.

ROS is used as the main software development tool for this project. It works as its name implies, a Robotic Operating System framework. Includes libraries and tools for sensory data acquisition, actuators control, and adds the possibility to run python or C++ based algorithms to enhance a robot’s performance. Figure 10 is generated using *rosgraph* a narrowed node topology diagram of our contribution to existing node infrastructure.

The /*camera/depth/color/points* topic provides raw depth-colored information from the Stereo Camera. /*full_data_scan* stores unprocessed laserscan data type from the LiDAR. /*tf_static* and /*tf* calculate the homogeneous transformation matrices between each sensor and the physical body of the robot. /*L_sonar dist* and /*R_sonar_dist* have the left and right Kalman filtered sonar distance measurement respectively. /*Neural Network_Exec* is the node in charge of receiving all the sensors output, formatting the distance points from the camera, and feed them all through the neural network. The output distance is published in the original /*scan* topic used by most OGM generator algorithms.

Most lightweight algorithms such as the sensor data acquisition programs run on the robot. Meanwhile, the OGM generators and the image processing unit which are graphic intensive tasks run on the remote PC or laptop.

#### 3.3.1. Remote Server (Raspberry Pi 4)

The ubuntu server 20.04 for the Raspberry is installed in a 32 Gb SD card as an image from the official raspberry website. ROS Noetic and its packages are downloaded and installed following the instructions from the ROS Wiki. The ROS packages to interpret the data from the LiDAR and to control the motors of the robots are downloaded as a standalone package from the Robotis website. Lastly, the drivers and instruction packages for the stereo camera were installed using the manual CMAKE method (compatible with ARM architecture of the raspberry) and a ROS Wrapper to interact with the rest of the ROS framework. While this last approach is somewhat experimental, it proved to work for our setup. The algorithm developed to interact with the sonars and obtain the median and the Kalman Filter used to preprocess the distance measurements also ran from the Raspberry and was coded in python 3.8.

The communication between the Turtlebot and Remote PC is conducted via SSH (Secure Shell) using an available wi-fi connection via static IP addresses on both ends. To wirelessly control the robot movements, there are two main options—using the Bluetooth controller included with the robot or use the *teleop* command from the main PC.

#### 3.3.2. Main Processing Unit (Toshiba Satellite L845 Laptop)

Similarly, to the remote server, Ubuntu 20.04 Desktop and ROS Noetic were installed to the laptop’s SSD to control the robot and have access to the robot’s sensory data without overloading the processing capacity of the raspberry. Python 3.8 was made default and ATOM served as a lightweight solution to code most of the algorithms. Libre Office was also installed to analyze live data and debugging.

### 3.4. Training and Running the Network

To implement the ANN, Keras, and TensorFlow libraries were used along with their sequential model which is a wrapper for creating models of a neural network with highly efficient algorithms. We tested the performance of our neural network with the RMSE score, it allowed us to determine how well-trained the NN is. The units of the error are the same as those of the variable is testing.

Initially, to train the network, distance data is sampled in batches of approximately 60 measurements (1 distance measurement per LiDAR degree). The size of the batch is determined automatically by the horizontal size of the image. This is to prevent results with missing information from the camera. There are two types of batches: True Distance and Detected Distance, these can be further analyzed in Table 3.

The True Distance batch is the actual true distance from the robot to the obstacles and Detected Distance is the perceived distance of each sensor. Since the Sonars do not have an angular resolution, the single distance measurement they obtain is used for all the angular positions within their range.

Please note from Table 3 how certain Detected LiDAR Distances are much bigger than the True Distance (See LiDAR detected distance for position $65{}^{{}^{\circ}}$, $66{}^{{}^{\circ}}$, $119{}^{{}^{\circ}}$, $120{}^{{}^{\circ}}$), this indicates the possible presence of glass in those positions. Meanwhile, since the sonars do not have an angular resolution, they obtain the closest distance to any obstacle within its angular range, hence all the values are the same for its region (See Detected Sonar Distance for positions $65{}^{{}^{\circ}}$ through $68{}^{{}^{\circ}}$ and positions $117{}^{{}^{\circ}}$ through $120{}^{{}^{\circ}}$). Finally, whilst the Camera also does not detect glass (See Detected Camera Distance for positions $65{}^{{}^{\circ}}$ and $66{}^{{}^{\circ}}$), it can detect out-of-view obstacles of the LiDAR that are not glass (See Camera detected distance for position $119{}^{{}^{\circ}}$ and $120{}^{{}^{\circ}}$) Each of these case scenarios are present in roughly one-third of the cases, respectively.

When taking measurements for both batches, the robot needs to remain still while the opaque tape is placed and removed. So, by driving the robot to specific indoor locations and sampling distance data of both batches, the data is obtained and preprocessed to diminish the loss value error of the NN output. The training dataset was obtained in a different physical location than the testing dataset.

**Figure 11:**
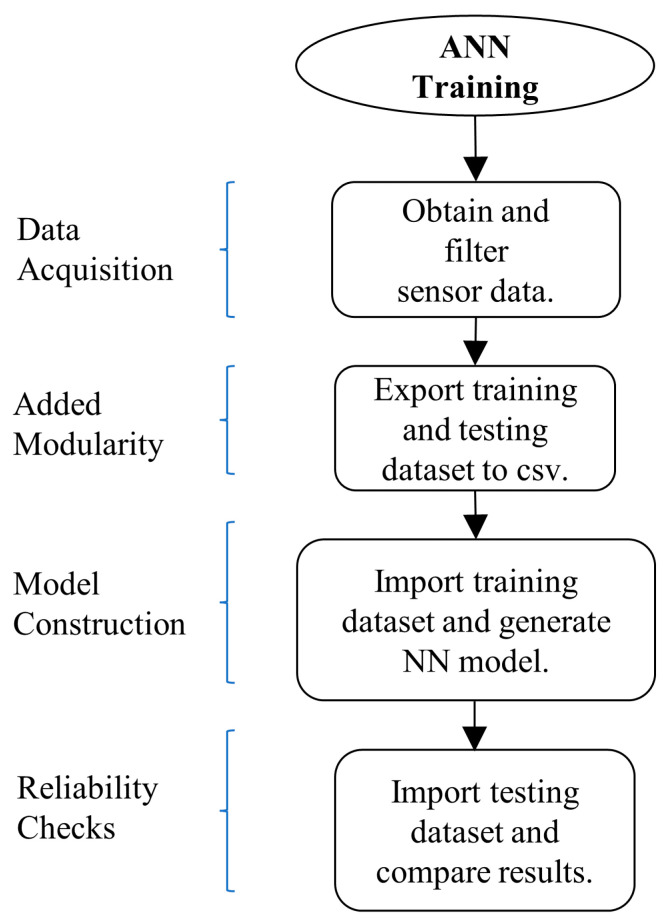
The neural network training stage.

Secondly, the formatted data is saved to a *.csv* file, from which it is taken by a standalone algorithm that creates the network, feeds the training data to the network, and generates a model with the weights of the network. In this stage, we fine-tune the network parameters to obtain the best result possible. In Figure 11 a diagram of these steps better presents each of the training stages.

**Figure 12:**
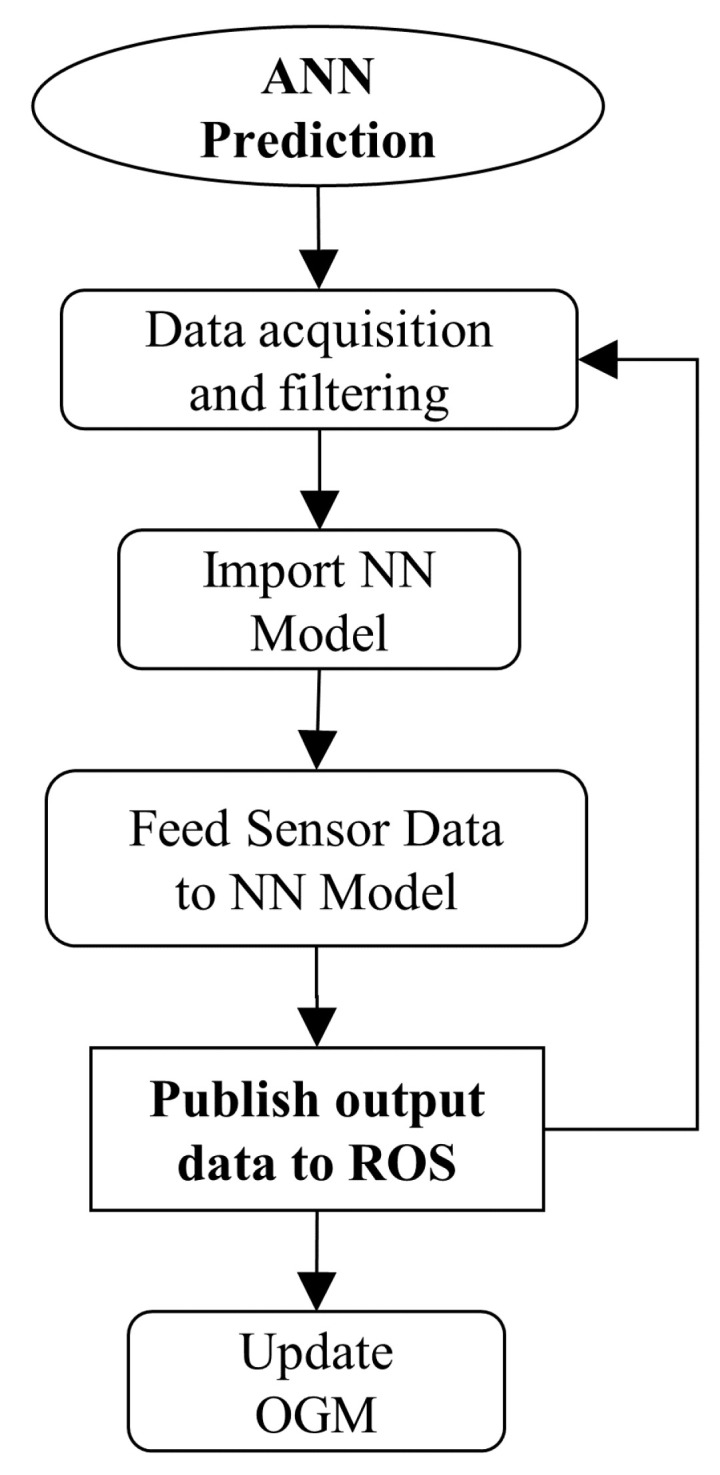
The neural network prediction working principle in which the data is obtained, fed to the NN, the distance output is generated and published into the ROS ecosystem for other services to use.

To implement the prediction stage of the NN, shown in Figure 12, as a functional part of the fusion algorithm, we launch the sensor data acquisition programs (in the Turtlebot3) which feeds its data via a wireless connection (Secure Shell) to a second program (running in a laptop) which filters it and makes it ready to be fed into the neural network as a *Laserscan* message for each sensor. Next, we import the model of the network and feed these sensor data to it. The last step is to publish the model output into the ROS ecosystem with a *Laserscan* message type, which will substitute the non-fused *Laserscan*.

Once in the prediction stage, the Raspberry processor heats up, diminishing the frequency with which the OGM is updated. The slowest frequency achieved was 0.2 Hz.

In Table 4 and Table 5, a resume of the NN optimization Algorithm and NN tuning parameters and structure is shown. The Inputs, Outputs, Hidden layers, and Neuron per Hidden layers determine the basic structure of the NN. This internal structure is initially defined using educated guests and later tuned based on following empirical methods. The Optimizer is the algorithm used to adjust the weights of each neuron. The activation function determines the state of the neuron, based on its numerical output. The number of Epochs indicates the number of times a complete dataset (with size Batch Size) moves across all layers of the network (forward and backwards). The loss function refers to the numerical value we seek to optimize (in this case it is minimized). The loss value number provides an indication of how well-trained the model is. The *kernel_initializer* indicates the statistical distribution that the initial weights of neurons should follow. Finally, the *BIAS_initializer* sets the initial numerical values of the weights each neuron should have.

Given the fact that we present two fusion strategies, multi-column tables are in place. These numerical values were fine-tuned using arbitrary methods, as there is no proper way of selecting them. There are plenty of tutorials online where default starting parameters can be found and adjusted individually for better results. These work as intended for our case scenario; however, they will probably have to be changed if the training dataset is changed as well. Some of the values are extremely sensitive to changes especially the ones that affect the training algorithm (ADAM optimizer). Notice how the NN that fuses data from three sensors is more complex than the NN that fuses data from two sensors.

## 4. Results

Three testing scenarios are presented to examine the performance of the data fusion algorithms with different types of variables.

The ground truth map was obtained using the data of the LiDAR having all obstacles taped with black non-reflective electrical tape. Objects that are not tall enough to be detected by the LiDAR were temporally substituted by others that have the same shape yet are tall enough to be detected by the LiDAR. After each ground truth map is created, all electrical tape was removed to conduct the experiments without tape in the testing scenarios. In Table 6 there is a legend explaining the color combination used in the OGM’s.

### 4.1. Scenario 1

**Figure 13:**
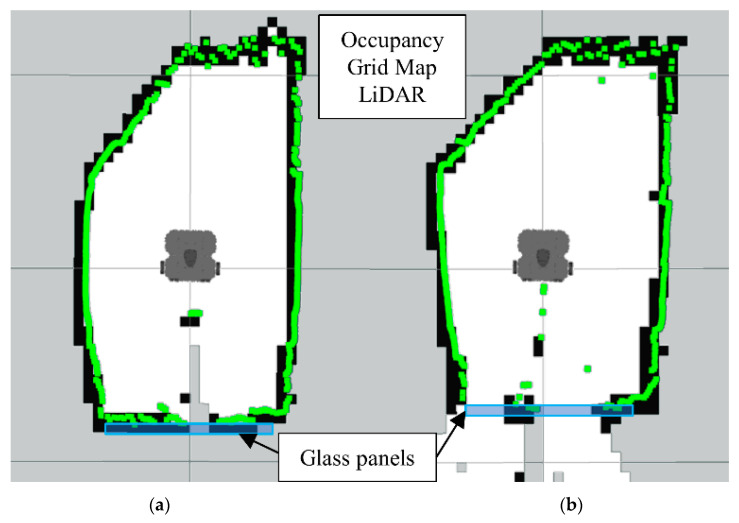
Occupancy grid maps showing the: (**a**) Ground truth (reference map); (**b**) LiDAR generated map.

First, we can analyze the results for the LiDAR-Sonar fusion strategy, starting with the OGM generated from just the LiDAR data. In Figure 13a we have the ground truth map, which is the reference map we can compare the rest of the results to. In Figure 13b we have just the LiDAR unable to detect the glass panels. Notice how the glass section appears transparent to the LiDAR resulting in an extremely inaccurate map.

**Figure 14:**
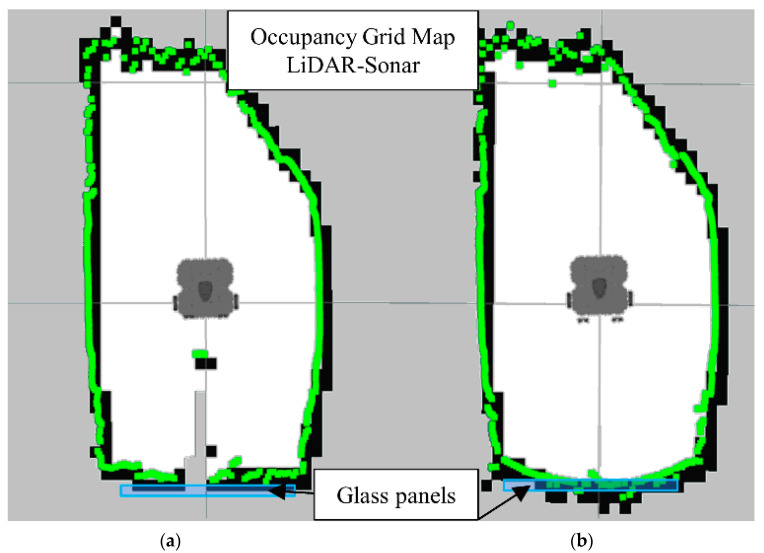
Occupancy grid maps showing the: (**a**) Ground truth (reference map); (**b**) LiDAR-Sonar fusion generated map.

**Figure 15:**
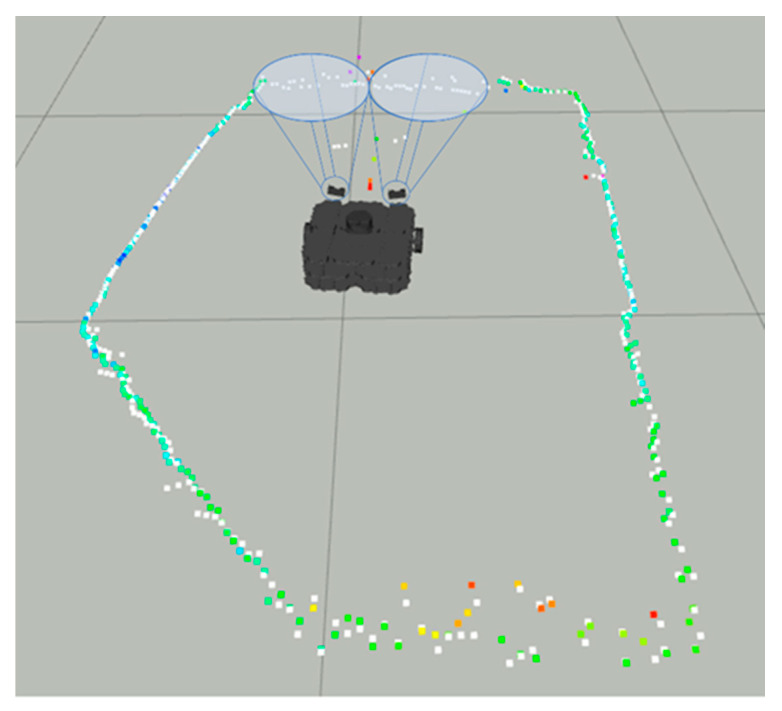
Sonar scanning region for LiDAR-Sonar fusion.

Figure 14b is the map generated using LiDAR-Sonar fusion data exclusively. In Figure 14a we have the ground truth. Notice how even though it can detect glass, the FoV is not large enough (See Figure 15) to detect a small obstacle placed directly in front of the robot.

Notice in Figure 15 the colored dots around the robot representing the original LiDAR Laserscan message, while the white dots represent the improved LiDAR after the LiDAR- Sonar fusion strategy.

**Figure 16:**
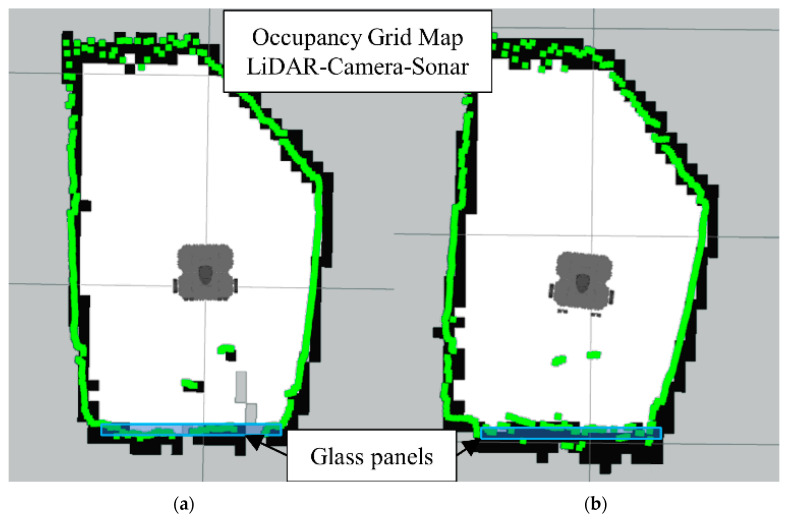
Occupancy grid maps showing the: (**a**) Ground truth (reference map); (**b**) LiDAR-Camara-Sonar fusion generated map.

In Figure 16b we have the occupancy grid maps generated by full sensors fusion scheme (LiDAR-Camera-Sonar). Also notice how the glass section from the LiDAR map is better represented in the data fusion scheme along with the smaller objects.

**Figure 17:**
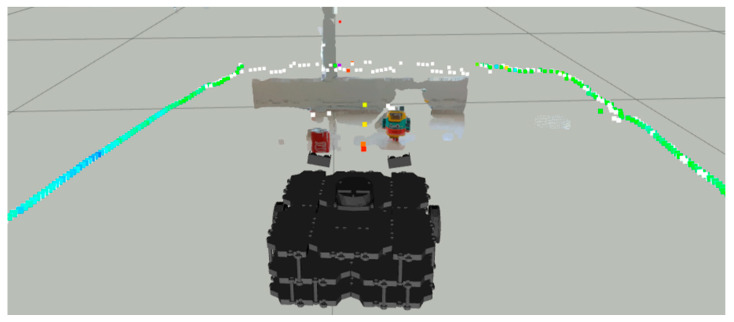
All sensors scanning region for LiDAR-Sonar-Camera fusion.

The fusion FoV for the LiDAR-Camera-Sonar approach can be seen in Figure 17. Note how the 3D image of the camera at ground level provides the definitive space at which the fusion takes place. White dots indicate a fused *Laserscan* message, and the colored dots indicate the pre-fusion LiDAR *Laserscan*.

### 4.2. Scenario 2

**Figure 18:**
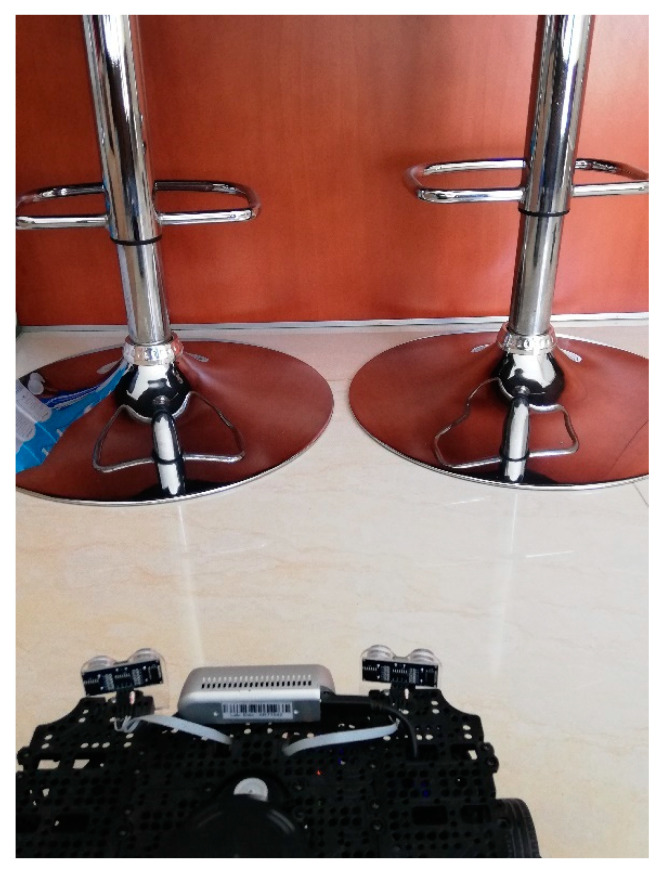
Point of interest of scenario 2.

In the second scenario, a combination of materials is tested. The main one is the aluminum base of the breakfast table chairs and the wooden panels. The base of the chairs has a conical base with a polished aluminum texture to it. Since the base of the chairs are a lot wider than the tube it supports them, the OGM generated by just the LiDAR is not accurate enough. In Figure 18 we have a picture of the real scenario for a better understanding of the physical layout.

**Figure 19:**
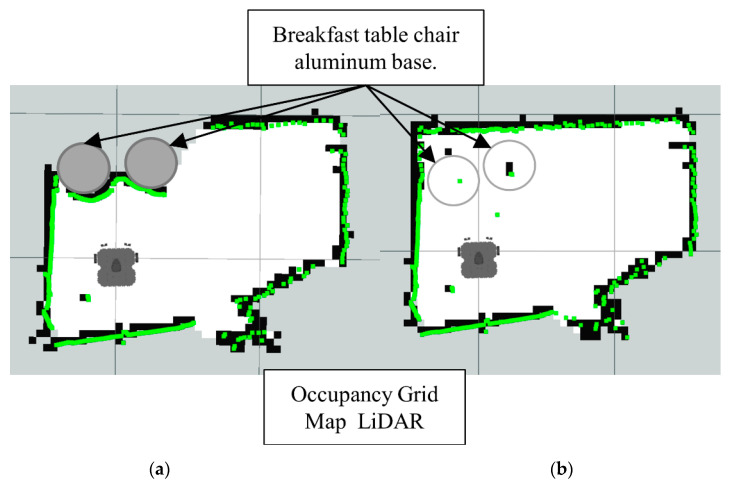
Occupancy grid maps showing the: (**a**) Ground truth (reference map); (**b**) LiDAR generated map.

**Figure 20:**
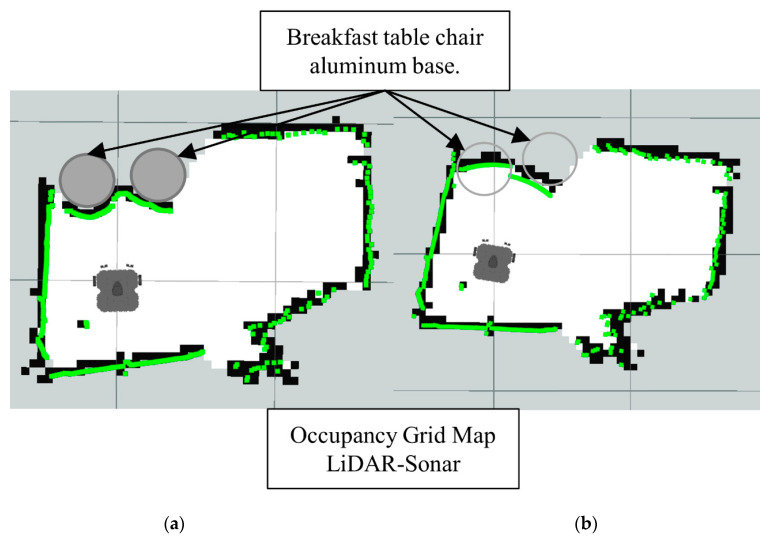
Occupancy grid maps showing the: (**a**) Ground truth (reference map); (**b**) LiDAR-Sonar fusion generated map.

Note how in Figure 19 the LiDAR alone cannot detect the full area of the breakfast table chair base. Meanwhile, in the Figure 20 the Lidar-Sonar fusion strategy shows this approach can effectively detect the obstacles, but it is not very accurate with the rounded sections of the obstacle.

**Figure 21:**
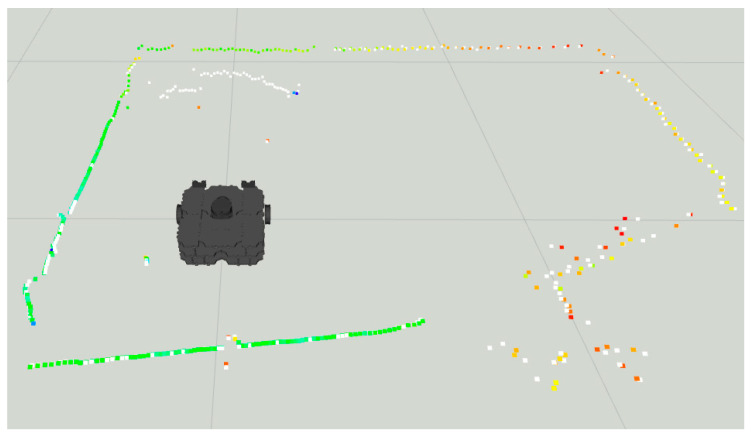
Distance readings pre and post fusion for LiDAR-Sonar fusion.

In Figure 21, the distance reading prior to the fusion strategy in colored dots vs. post fusion in white.

**Figure 22:**
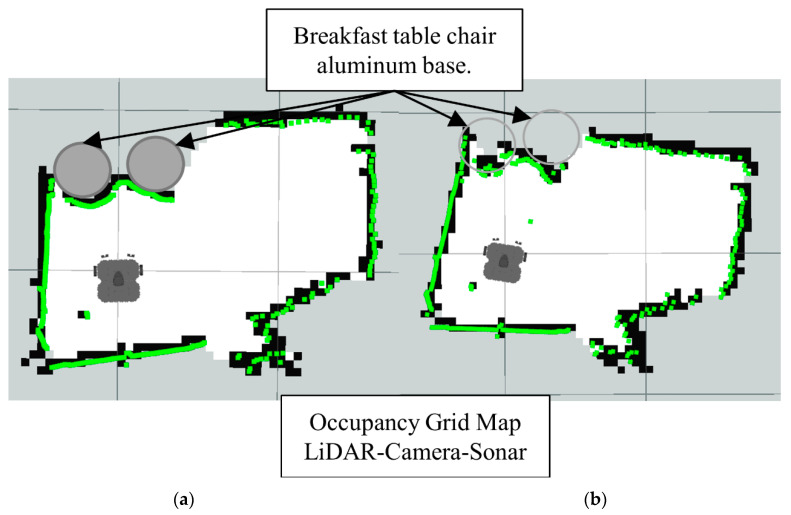
Occupancy grid maps showing the: (**a**) Ground truth (reference map); (**b**) LiDAR-Camara-Sonar fusion generated map.

**Figure 23:**
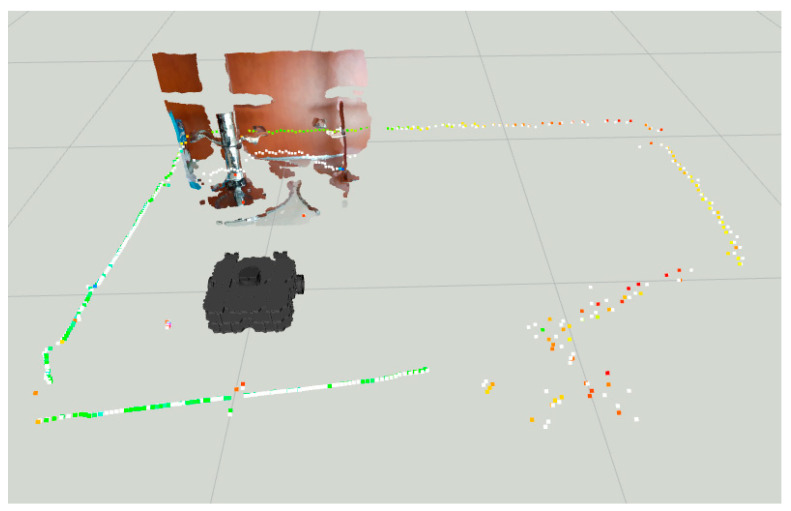
All sensors scanning region for LiDAR-Sonar-Camera fusion.

Finally, the LiDAR-Camera-Sonar approach in Figure 22 has the most accurate representation of the obstacles. Also, in Figure 23 the *laserpoints* before and after fusing the data provide a clearer representation of the overall improvement in obstacle localization.

### 4.3. Scenario 3

**Figure 24:**
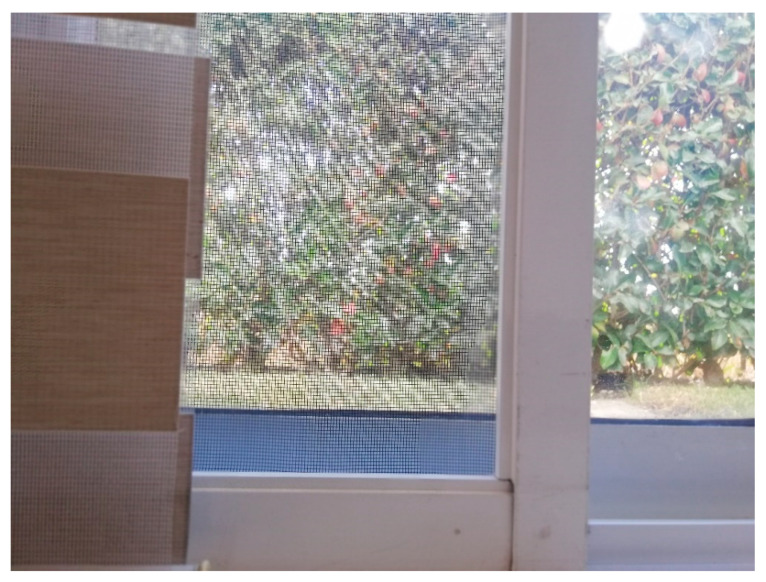
Different Materials tested in scenario 3.

**Figure 25:**
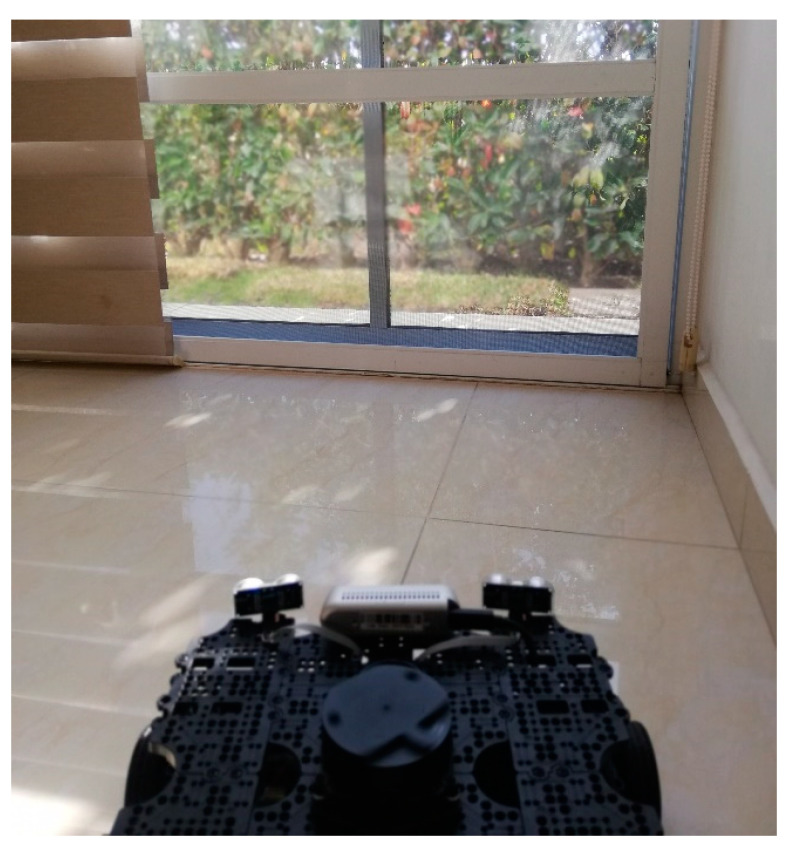
Point of interest of scenario 3.

In the third and last scenario a combination of meshes are compared to show the effectiveness of the method used to detect the obstacles. In Figure 24 and Figure 25 we present the four different obstacles to detect. In the left side of the image a mesh window curtain. In the center we have an insect mesh trap door, and to the right of the image we have an unobstructed view of an outdoor environment through transparent glass. Please notice all the painted aluminum partitions.

**Figure 26:**
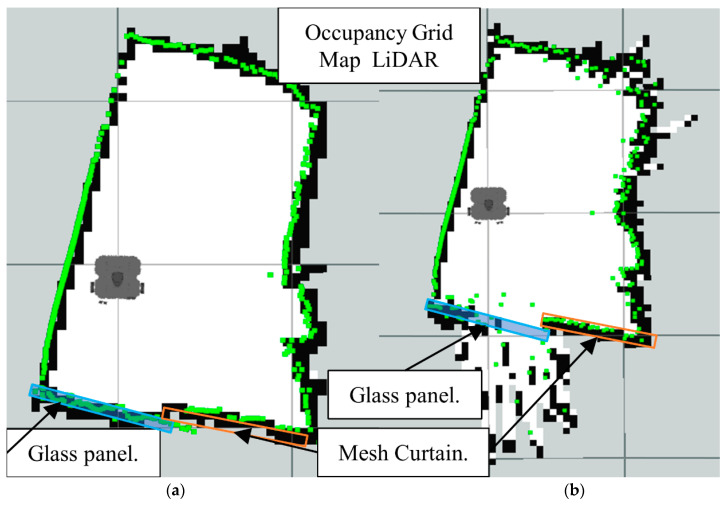
Occupancy grid maps showing the: (**a**) Ground truth (reference map); (**b**) LiDAR generated map.

**Figure 27:**
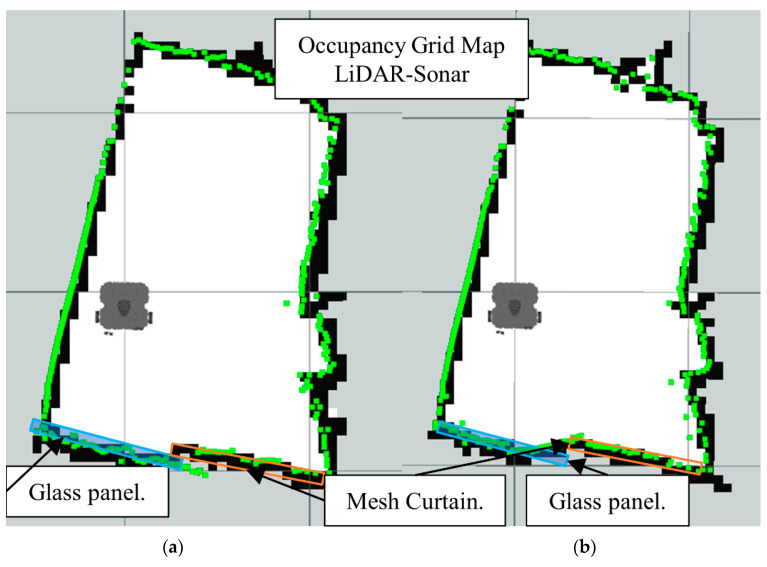
Occupancy grid maps showing the: (**a**) Ground truth (reference map); (**b**) LiDAR-Sonar fusion generated map with glass panel.

**Figure 28:**
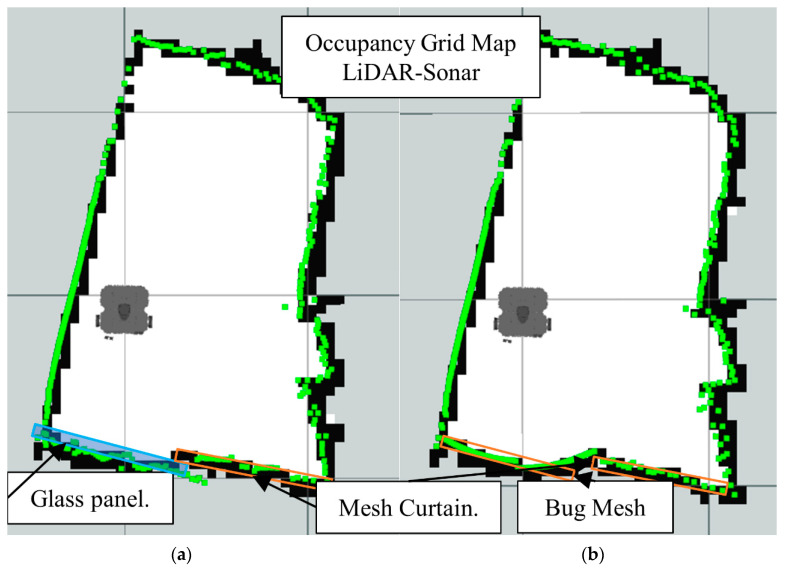
Occupancy grid maps showing the: (**a**) Ground truth (reference map); (**b**) LiDAR-Sonar fusion generated map with insect door mesh in front of the glass.

**Figure 29:**
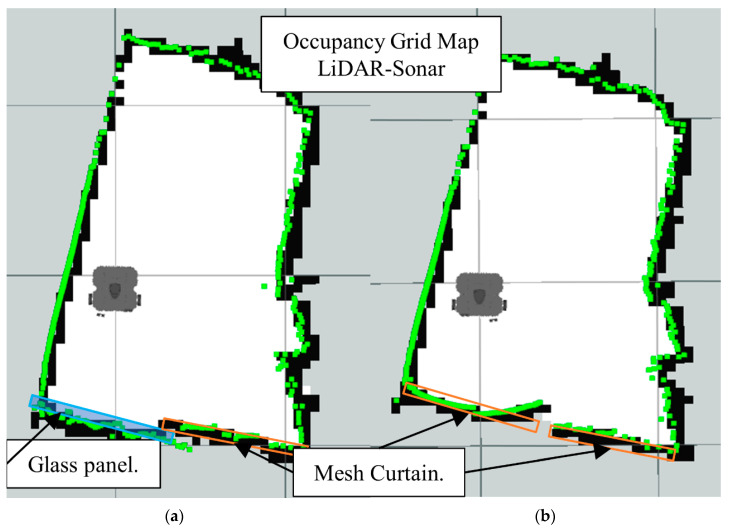
Occupancy grid maps showing the: (**a**) Ground truth (reference map); (**b**) LiDAR-Sonar fusion generated map with dense curtain mesh in front of the glass.

Figure 26 shows the LiDAR inability to detect the glass panel and other obstacles with opposing lighting conditions, while Figure 27, Figure 28 and Figure 29, present the OGM generated with the LiDAR-Sonar fusion scheme while detecting different types of obstacles. Figure 27 shows the performance while testing for glass, in Figure 28 we test for bug mesh and Figure 29 for curtain mesh.

**Figure 30:**
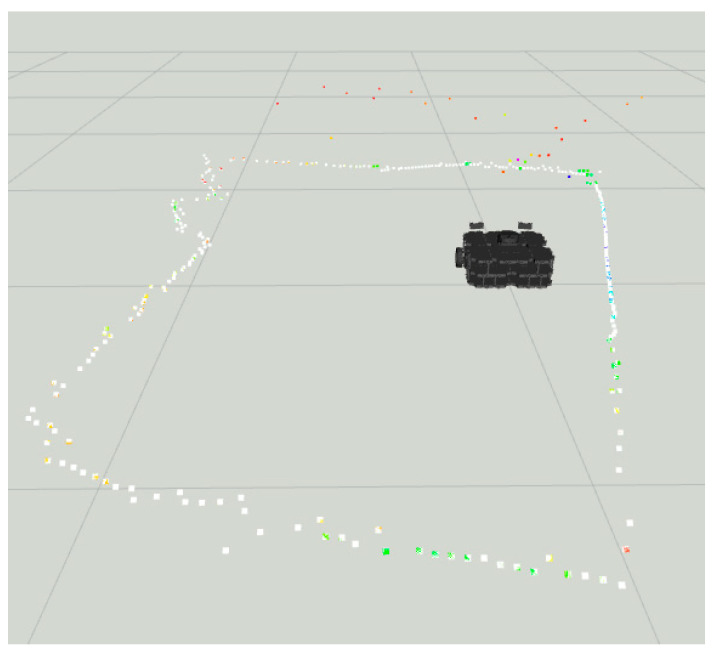
Pre and post fusion readings for LiDAR-Sonar fusion using curtain mesh as main obstacle.

In Figure 30, we have the before and after fusion *Laserscan* readings with the mesh curtain as the main obstacle.

**Figure 31:**
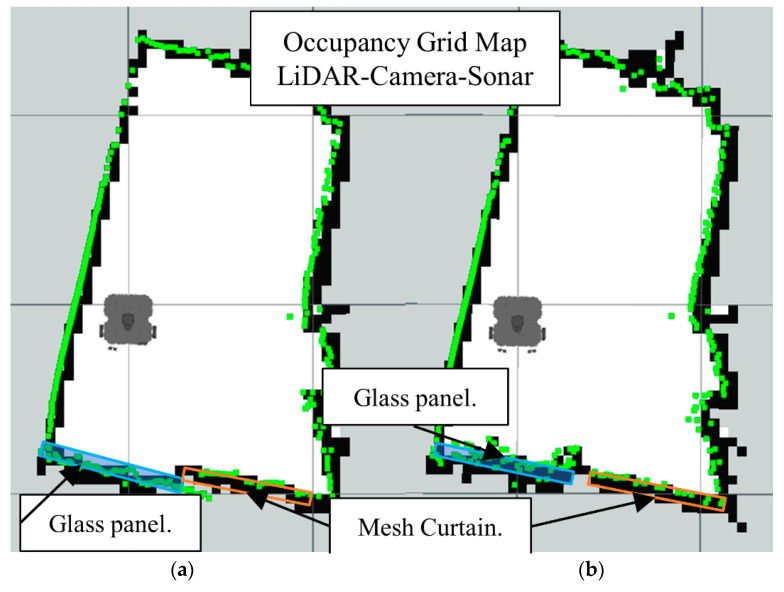
Occupancy grid maps showing the: (**a**) Ground truth (reference map); (**b**) LiDAR-Camara-Sonar fusion generated map with glass as main obstacle.

**Figure 32:**
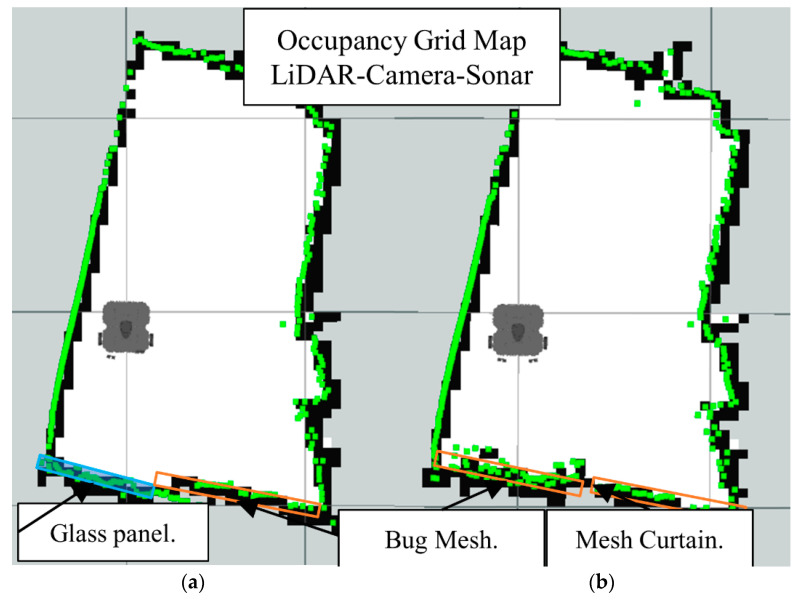
Occupancy grid maps showing the: (**a**) Ground truth (reference map); (**b**) LiDAR-Camara-Sonar fusion generated map with insect/bug mesh as main obstacle.

**Figure 33:**
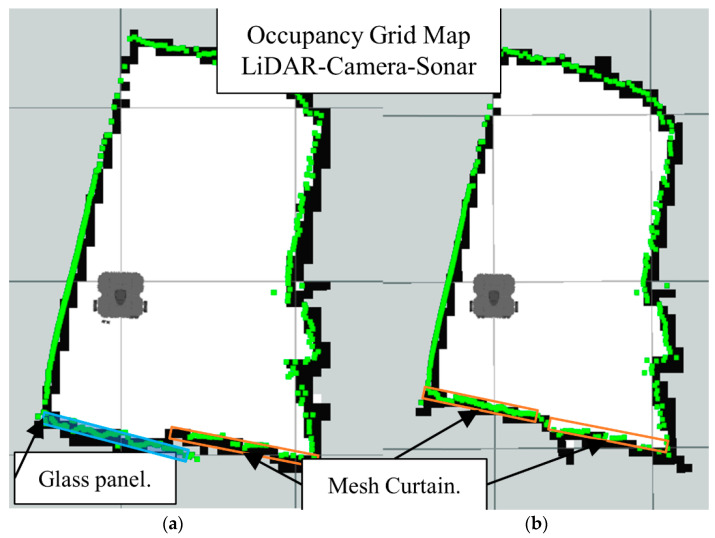
Occupancy grid maps showing the: (**a**) Ground truth (reference map); (**b**) LiDAR-Camara-Sonar fusion generated map with mesh curtain as main obstacle.

Now we present the results of the LiDAR-Sonar-Camera fusion strategy for the same obstacle setup starting with glass obstacle in Figure 31, insect mesh in Figure 32 and curtain mesh in Figure 33.

**Figure 34:**
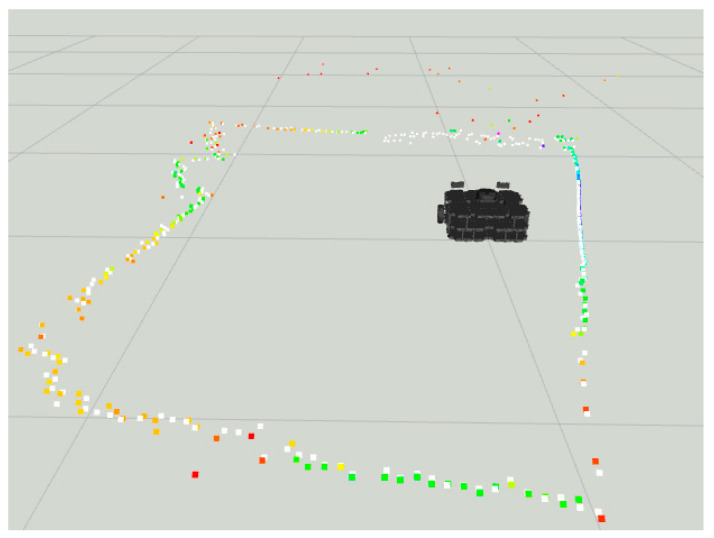
Pre and post fusion readings for LiDAR-Sonar-Camera fusion using bug mesh as main obstacle.

In Figure 34 we have the distance readings before and after applying the fusion strategy. The RMSE score is the error measure selected to mathematically represent the accuracy of the maps w.r.t the ground truth map. In Table 7 the RMSE value of the LiDAR explains the lackluster performance in the occupancy grid map of the LiDAR at detecting glass. Also, the averaged RMSE for the fusion strategies is between two and three centimeters in error as seen in Table 7.

**Figure 35:**
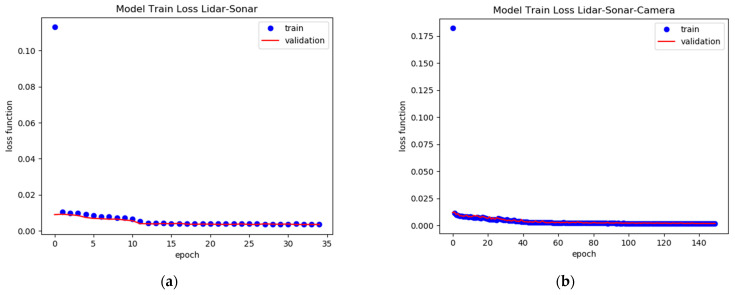
Model Validation Loss Function: (**a**) LiDAR-Sonar fusion; (**b**) LiDAR-Sonar-Camera fusion.

The training loss and the validation loss graphs in terms of loss per epoch show a decreasing tendency at roughly the same level. This indicates a balanced learning procedure shown in Figure 35.

**Figure 36:**
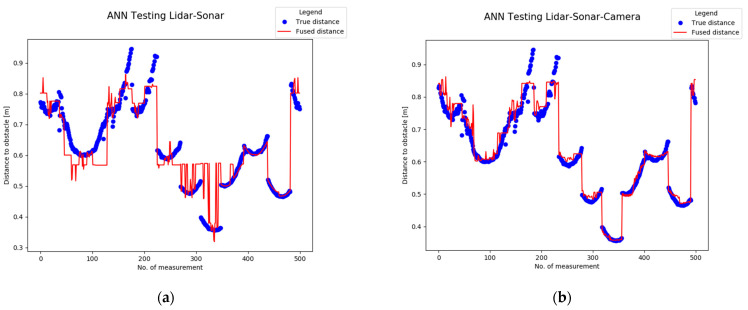
Analytical performance evaluation of the neural network by comparing the ground truth distance and fused distance: (**a**) LiDAR-Sonar model; (**b**) LiDAR-Sonar-Camera model.

Next, the ground truth (True Distance) versus the Fused distance in Figure 36 indicates the neural networks are properly trained, with a minimum error between the input and output. Notice the increased amount of training points in the LiDAR-Camera-Sonar network compared to the smaller and simpler LiDAR-Sonar network.

Figure 36a,b may look similar however each one has a different response to the input data. In the case of the LiDAR-Sonar (Figure 36a) the fused distance output in red is less accurate. Meanwhile, Figure 36b has a more accurate representation of the obstacles.

**Figure 37:**
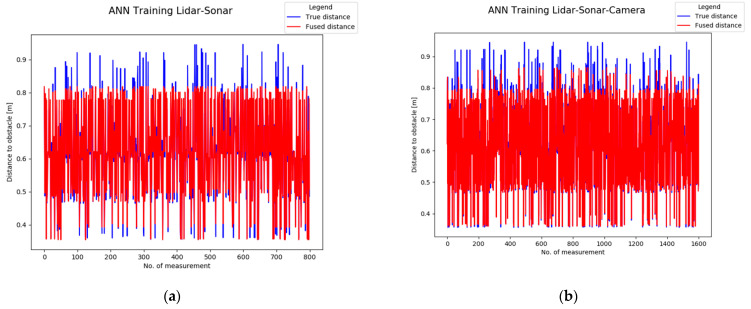
Validation of neural network with randomized data for: (**a**) LiDAR-Sonar model; (**b**) LiDAR-Sonar-Camera model.

In Figure 37a,b by randomizing the order at which the distance to the obstacle for the training stage is imported, it shows that both networks are learning and not memorizing during the training phase.

## 5. Conclusions

LiDARs are widely used in the field of mobile robotics due to their high accuracy, fast data acquisition, and ease of use in a mobile environment. However, 2D units lack the ability to detect obstacles outside their single plane FoV. Furthermore, due to the sensing strategy implemented, they are not able to detect glass surfaces reliably. So, a mobile robot data fusion approach was implemented to use the best features of multiple sensing technologies in a triple sensor setup. By using the capability of ultrasonic sensors to detect glass, the ability to accurately detect objects in a 3D environment of the Stereo Camera, and the spatial resolution of the LiDAR we improved the LiDAR capability to detect glass and additional objects outside the FoV of a regular 2D LiDAR.

Two fusion algorithms were tested: LiDAR-Sonar and LiDAR-Camera-Sonar and compared with state-of-the-art LiDAR generated Occupancy Grid Maps. The RMSE values for the data fusion algorithms are consistent with the quality of the occupancy grid maps generated. The NN performed better at detecting glass than the LiDAR with both algorithms. The results between both data fusion strategies show that while the camera is not that relevant at detecting glass it is able to accurately detect 3D obstacles underneath the FoV of the LiDAR. This very last feature represents an improvement over the Sonar-Lidar sensor setup. While some authors have successfully dealt with such a problem, we present an alternate solution that is usually used to tackle a different kind of problem, where way more powerful hardware is required. In this case, the solution was implemented in a piece of mobile robotic hardware not capable of carrying around a laptop.

Lastly, future work includes exploring the effect of different lighting conditions, sound levels, and wind velocity in an outdoor environment. Moreover, additional testing of different data fusion methods to have as a baseline model in more complex testing scenarios can prove to be helpful. Also, it could be interesting to test the same setup with hardware processing improvements for a smoother operation, especially in the LiDAR-Camera-Sonar strategy. This final suggestion can have a great impact, as we suspect it will significantly increase the processing ability of the robot when detecting obstacles at longer distances.

## Figures and Tables

**Table 1:** Kalman Filter variables and initial values.

Variable Name	Representation	Initial Value
State vector	$x_{k}\in {\mathbb{R}}^{n}$	-
Feedback	$u_{k}\in {\mathbb{R}}^{l}$	-
Measurement	$z_{k}\in {\mathbb{R}}^{m}$	-
Constant Matrix State	$F\in {\mathbb{R}}^{nxn}$	[1]
Feedback	$B\in {\mathbb{R}}^{nxl}$	[0]
Measurement	$H\in {\mathbb{R}}^{mxn}$	[1]
Noise covariance of matrix	Q	0.1
Covariance measurement.	R	0.4
A priori state estimation	${\hat{x}}_{k}{}^{\left(-\right)}$	[1]
Covariance matrix a priori	$P_{k}{}^{\left(-\right)}$	[1]

**Table 2:** Main Data fusion methods and techniques classification.

No. Criteria	Detail	Topology
**1.**	Relation between the input data sources.	Complementary.
		Redundant.
		Cooperative.
**2.**	Type of employed data	Raw measurements.
		Signals.
		Characteristics or decisions.
**3.**	Architecture type	Centralized.
		Decentralized.
		Distributed.

**Table 3:** ANN training dataset example.

Angular Position of Distance Measurement	True Distance (Using Opaque Masking Tape)	Detected Distance (Not Using Opaque Electric Tape)		
	LiDAR	LiDAR	Sonar	Camera
$65{}^{{}^{\circ}}$	20 cm	200 cm	22 cm	190 cm
$66{}^{{}^{\circ}}$	23 cm	190 cm	22 cm	180 cm
$67{}^{{}^{\circ}}$	25 cm	24 cm	22 cm	21 cm
$68{}^{{}^{\circ}}$	30 cm	31 cm	22 cm	23 cm
**…**	…	…	…	…
$117{}^{{}^{\circ}}$	39 cm	38 cm	40 cm	38 cm
$118{}^{{}^{\circ}}$	40 cm	39 cm	40 cm	40 cm
$119{}^{{}^{\circ}}$	41 cm	120 cm	40 cm	42 cm
$120{}^{{}^{\circ}}$	43 cm	170 cm	40 cm	43 cm

**Table 4:** Adam Configuration Parameters.

ADAM Parameter	Description	Value	
		Lidar-Camera-Sonar	Lidar-Sonar
α	Step size	−0.008	−0.0015
$\beta_{1}$	Exponential decay rate for 1st moment estimate	$\frac{2}{epochs}=0.013$	$\frac{2}{epochs}=0.057$
$\beta_{2}$	Exponential decay rate for 2nd moment estimate	$\frac{2}{epochs}=0.013$	$\frac{2}{epochs}=0.057$
f(θ)	Stochastic objective function with parameters θ	MSE	
$\theta_{0}$	Initial parameter vector	Zeros	
$m_{0}$	Initialize 1st moment vector	Zeros	
$v_{0}$	Initialize 2nd moment vector	Zeros	
t	Initialize timestep	0	

**Table 5:** Tuning parameters of the NN.

ANN Parameter	LiDAR-Sonar-Camera	LiDAR-Sonar
Inputs	3	2
Outputs	1	1
Hidden Layers	50	6
Neuron per Hidden Layer	80	60
Optimizer	Adam	Adam
Activation Function	ReLu	ReLu
Epochs	150	35
Loss function	MSE	MSE
Metric of Loss function	mse	mse
Batch size	4	2
kernel_initializer	he_uniform	he_uniform
BIAS_initializer	zeros	zeros

**Table 6:** OGM color legend combination.

Color	Legend
Green	Live sensory data
Blue	Glass panels
Black	Known obstacles
White	Empty area
Gray	Unknown area

**Table 7:** RMSE between fusion strategies and LiDAR vs. Ground Truth.

Data	LiDAR-Sonar	LiDAR-Sonar-Camera
Train Dataset	0.03289 m	0.026143 m
Test Dataset	0.03567 m	0.029696 m
LiDAR Dataset	2.12175 m	2.121759 m
